# Transport of COVID-19 and other highly contagious patients by helicopter and fixed-wing air ambulance: a narrative review and experience of the Swiss air rescue Rega

**DOI:** 10.1186/s13049-020-00734-9

**Published:** 2020-05-14

**Authors:** Roland Albrecht, Jürgen Knapp, Lorenz Theiler, Marcus Eder, Urs Pietsch

**Affiliations:** 1 Department of Anaesthesiology and Intensive Care Medicine, Cantonal Hospital St. Gallen, Rorschacher Strasse 95, 9007 St. Gallen, Switzerland; 2 Swiss Air Rescue, Rega, Zurich, Switzerland; 3 Department of Anaesthesiology and Pain Medicine, Inselspital, Bern University Hospital, University of Bern, Bern, Switzerland

**Keywords:** HEMS, Air ambulance, COVID-19, Highly contagious patient, Transport

## Abstract

**Background:**

The current COVID-19 pandemic highlights the challenges air ambulance services are facing when transporting highly infectious patients for several hours in enclosed spaces. This overview provides an example of a standard operating procedure (SOP) for infection prevention measures in HEMS missions during the COVID-19 pandemic. Furthermore, we describe different methods used by several organizations in Europe and the experience of the Swiss air rescue organization Rega in transporting these patients.

Possible benefits of the use of small patient isolation units (PIU) are discussed, including the fact that accompanying medical personnel do not need to wear personal protective equipment (PPE) during the transport but can still maintain full access to the patient. Rega has developed and patented its own PIU. This device allows spontaneously breathing or mechanically ventilated patients to be transported in pressurized jet cabins, small helicopters and ambulance vehicles, without the need to change between transport units. This PIU is unique, as it remains air-tight even when there is a sudden loss of cabin pressure.

**Conclusion:**

A wide variety of means are being used for the aeromedical transport of infectious patients. These involve isolating either the patient or the medical crew. One benefit of PIUs is that the means of transport can be easily changed without contaminating the surroundings and while still allowing access to the patient.

## Background

During the current Coronavirus (COVID-19) pandemic, ground and air rescue, rotary-wing (HEMS) and fixed-wing (AEMS) emergency medical services are faced with unprecedented challenges. While there are several recommendations for protective measures during in-hospital emergency procedures and tracheal intubation, there is insufficient guidance regarding the prehospital setting [1]. Moreover, so far there are no published guidelines on how to safely transfer COVID-19 patients.

Unfortunately, the disease transmission risk for aeromedical crew members is higher than for in-hospital healthcare providers, largely due to information, resources and space being very limited in the prehospital setting, and aerosol-generating procedures such as airway management and ventilation being one of the main tasks. Different approaches are used to prevent transmission from patients with highly contagious infections during air transport. To address these issues we have reviewed current concepts in aeromedical transport in Europe, and we present the experience and recent recommendations of the Swiss Air Rescue (Rega) for COVID-19 and aeromedical transport.

### Recommendations for HEMS missions during the COVID-19 pandemic

Every air rescue provider should develop precise SOPs for the use and handling of PPE during HEMS missions. Especially during the current pandemic, with high numbers of infected patients (and individuals with unknown COVID-19 status), strict adherence to these standards on every mission is essential. Simulation training of these special measures to prevent infection is highly recommended for every crew member, as this is known to improve adherence to the SOPs [2, 3].

Table 1 provides an example of an SOP for infection prevention measures in primary (and secondary) HEMS missions during the COVID-19 pandemic, as used by Rega.

**Table 1:** Sample SOP “Infection prevention measures for HEMS missions during the COVID-19 pandemic”

CDR	• Maintain a distance of at least two meters from the patient. If this is possible, wearing PPE is not mandatory. This applies to the site where the HEMS crew is operating, as well as during the flight. • Join the medical crew in the emergency department or intensive care unit only in exceptional cases. If so, wear the same personal protective equipment as the medical crew.
MCM/HCM	• Wear examination gloves, a filtering facepiece class 2 mask (FFP2/3), and goggles for eye protection on every mission. Carry a bottle of hand disinfectant with you. • If possible, maintain a distance of > 2 m from the patient during initial contact and while checking for COVID-19 risk factors • Put a surgical mask over the patient’s mouth and nose or (depending on the clinical condition) a tight-fitting non-rebreather oxygen mask with adequate oxygen flow and a surgical mask over the exhalation valves. • Avoid aerosol-generating procedures such as non-invasive ventilation (NIV), high-flow oxygen therapy, tracheal suction, or nebulization of medications. • When planning for aerosol-generating procedures (mechanical ventilation, airway management, oral suction, cardiopulmonary resuscitation, etc.), put on a protective gown (if time permits) • Ventilator handover (e.g., emergency unit to Rega): - Avoid unnecessary respirator circuit disconnections - Set FiO_2_ to 1.0 - Check sedation/analgesia and relaxation (consider bolus administration if necessary) - Prepare a generic self inflating bag with flexible tube extension and airway filter, plus reservoir bag including attached oxygen supply - Switch the Rega ventilator device to standby mode - Switch off the ventilator device of the emergency unit - Clamp the tube between the patient and the filter - Connect Rega ventilation tubes with airway filter - Unclamp the tube - Switch on the Rega ventilator system - Verify correct positioning of the tube by using the CO2 curve on the Rega monitor and comparing ventilation settings, in particular the ventilation pressure (avoid auscultation unless absolutely necessary)

*CDR* Commander, *MCM* doctor, *HCM* paramedic

During the COVID-19 pandemic, Rega has not used closed suction catheters on primary missions or for transfers between hospitals (secondary transport), and their use is avoided even if they are pre-installed, due to the risk of aerosol production from mobile suction units. Furthermore, most patients with COVID-19 (without bacterial superinfection) have only minor production of bronchial secretions.

### Secondary transport of COVID-19 patients

Air rescue providers use different concepts for the secondary transport of COVID-19 patients: open transport systems that allow direct patient management through the medical crew wearing PPE (e.g., FFP2/3 mask, goggles or face shield, gloves and protective gown) throughout the transport [4], or closed transport systems (so-called air transport isolator systems) that were originally developed for the fixed-wing transport of patients with other highly contagious and dangerous diseases, such as viral hemorrhagic fever.

**Fig. 1:**
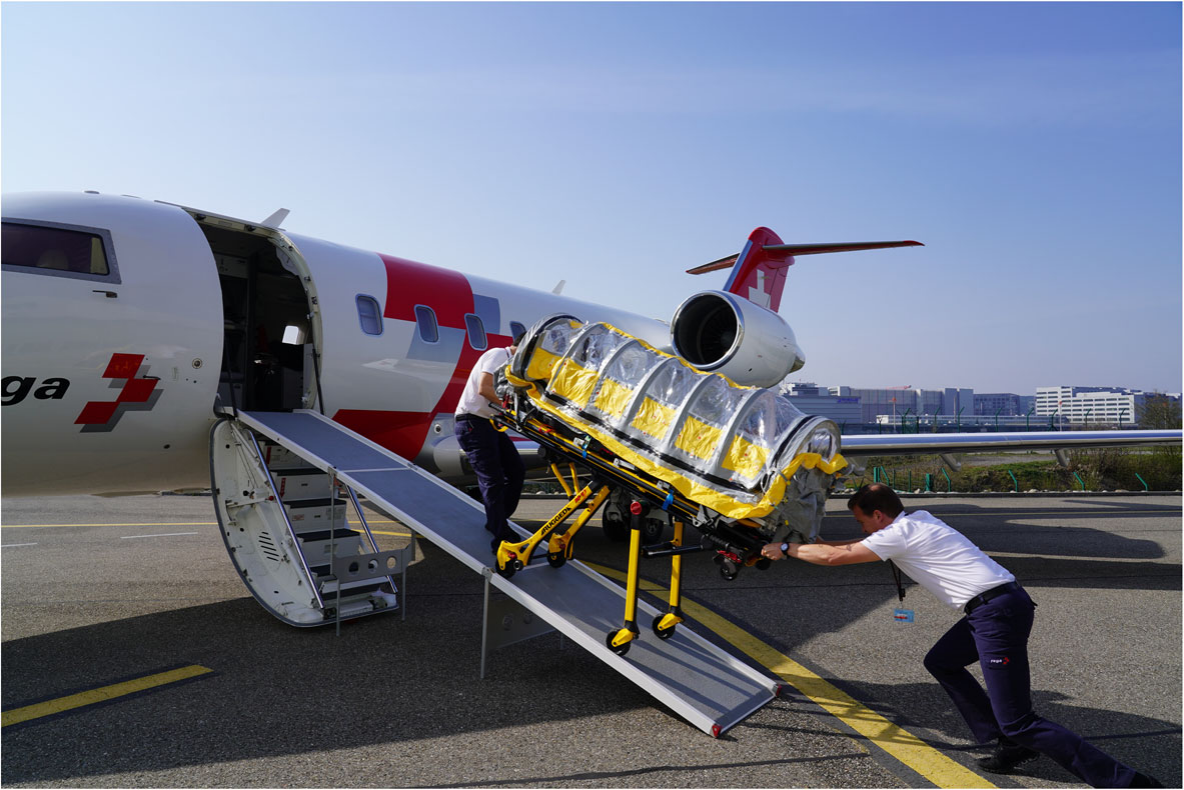
Rega PIU AEMS

There are two basic designs of these isolator systems: closed patient isolation units (PIU), which separate the patient from the medical crew (e.g., EpiShuttle®, EpiGuard HQ, Oslo Norway); Stretcher-Air Transport Isolators (S-ATI), and the larger Trexler Air Transport Isolator (T-ATI; Trexler Air Transport Isolator, UK, United Kingdom and the Rega PIU (Fig. 1). Alternatively, there are open systems which provide a portable isolation facility large enough for both the patient and attending medical staff wearing personal protective equipment (e.g., multi patient transport unit, the Containerized Biological Containment System, CBCS, Phoenix Arizona, USA). In the case of COVID-19, open PIUs do not offer an additional benefit.

Table 2 provides an overview of current concepts used by different air rescue providers in Europe for transporting COVID-19 patients (as of April 5th, 2020; only providers with verified information are listed). Although the authors have made considerable efforts to collect information about transported COVID-19 patients and their operational backgrounds, the table is not exhaustive. Additional patients might have been transported by additional operators or in different aircraft; operational orders may have been modified by the date of publication, etc. Furthermore, not all operators provided the requested information, and there are certainly additional operators who might have transported COVID-19 patients. Nonetheless, the table provides insight into patients transported and the range of operators, as well as into the operational infection control mechanisms involved. At the moment, there is no pan-European register for reporting numbers of transported COVID-19 patients.

**Table 2:** Overview of transport concepts for secondary missions for COVID-19 patients

Germany	German Armed Forces	Fixed-wing (A310 MedEvac and A400M): ventilated patients and non-ventilated patients/stretchers in the A310 MedEvac; open cabin, medical crew wearing PPE
	DRF Air Rescue	**Helicopter (H145):** only ventilated patients, medical crew wearing PPE; soon closed PIU (EpiShuttle®)
	ADAC	**Helicopter (H145):** only ventilated patients, medical crew wearing PPE
Switzerland	REGA	**Fixed-wing (CL650):** Rega PIU **Helicopter (H145, AW109):** medical crew wearing PPE, Rega PIU
	Air Zermatt Air Glacier	**Helicopter (Bell 429):** medical crew wearing PPE
Austria	ÖAMTC	Only ground-based transportation of confirmed cases
Italy	South Tyrol Tuscany Pegaso 1–3	Only ground-based transportation of confirmed cases **Helicopter (AW 169)** medical crew wearing PPE
France		Preferentially ground-based transportation of confirmed cases
Spain		Preferentially ground-based transportation of confirmed cases
United Kingdom		Preferentially ground-based transportation of confirmed cases
Poland		Only ground-based transportation of confirmed cases
Hungary		Only ground-based transportation of confirmed cases
Romania		Only ground-based transportation of confirmed cases
Slovakia		Only ground-based transportation of confirmed cases
Czech Republic		Only ground-based transportation of confirmed cases
Norway	Norwegian Air Ambulance	**Helicopter (H145 and AW 139):** closed PIU (EpiShuttle®)
	Royal Norwegian Air Force	**Helicopter (Sea King and Bell 412):** closed PIU (EpiShuttle®)
Sweden		Preferentially ground-based transportation of confirmed cases
Denmark		Preferentially ground-based transportation of confirmed cases
Netherlands		**Helicopter (H145):** medical crew wearing PPE

*PIU* patient isolation unit, *PPE* personal protective equipment

For secondary missions that often last several hours, it must be taken into account that working in full PPE during the transport of COVID-19 patients – especially in fixed-wing ambulances or helicopters – is very exhausting and physically stressful for the medical team, which may result in medical errors [3]. Furthermore, close attention must be paid to every movement, in order to avoid accidental disease transmission (e.g., by touching your own face with contaminated gloves). Therefore, for the aeromedical transport of patients with possible or confirmed COVID-19 on secondary missions (fixed-wing ambulances or long-lasting secondary HEMS missions), the use of a PIU offers substantial benefits, in spite of the higher costs and logistical requirements [5].

**Fig. 2:**
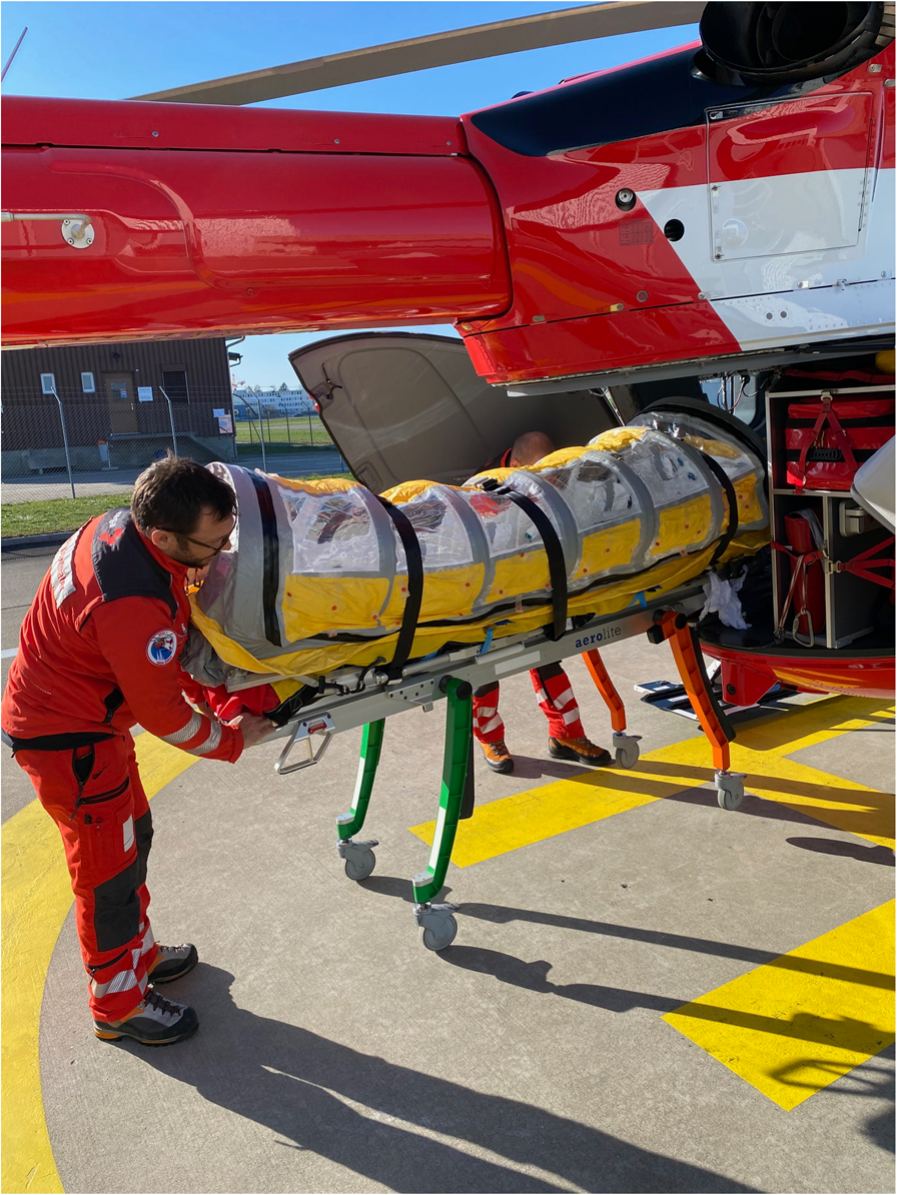
Rega PIU HEMS: Loading or unloading of the patient in the PIU

Closed PIUs also allow patients to be airlifted faster, as fixed-wing ambulances or helicopters do not require additional decontamination between transports (Fig. 2). Another advantage of using small PIUs is the easy transfer from an airplane to an ambulance or rescue helicopter, or vice versa. Thus, all teams involved in transporting these patients can be effectively protected, and the available FFP2/3 respirators can be reserved for use in hospitals. Both commonly used, small, closed PIUs (EpiShuttle® and Rega PIUs) offer these advantages.

However, at present the overall number of PIUs available for Rega is actually limited because they are normally manufactured in Italy (Lombardy). With regard to possible future epidemics or pandemics, to keep a minimum number of PIUs on hand for use in the case of a pandemic might be reasonable.

### Setup and our experience with the Rega PIU

**Fig. 3:**
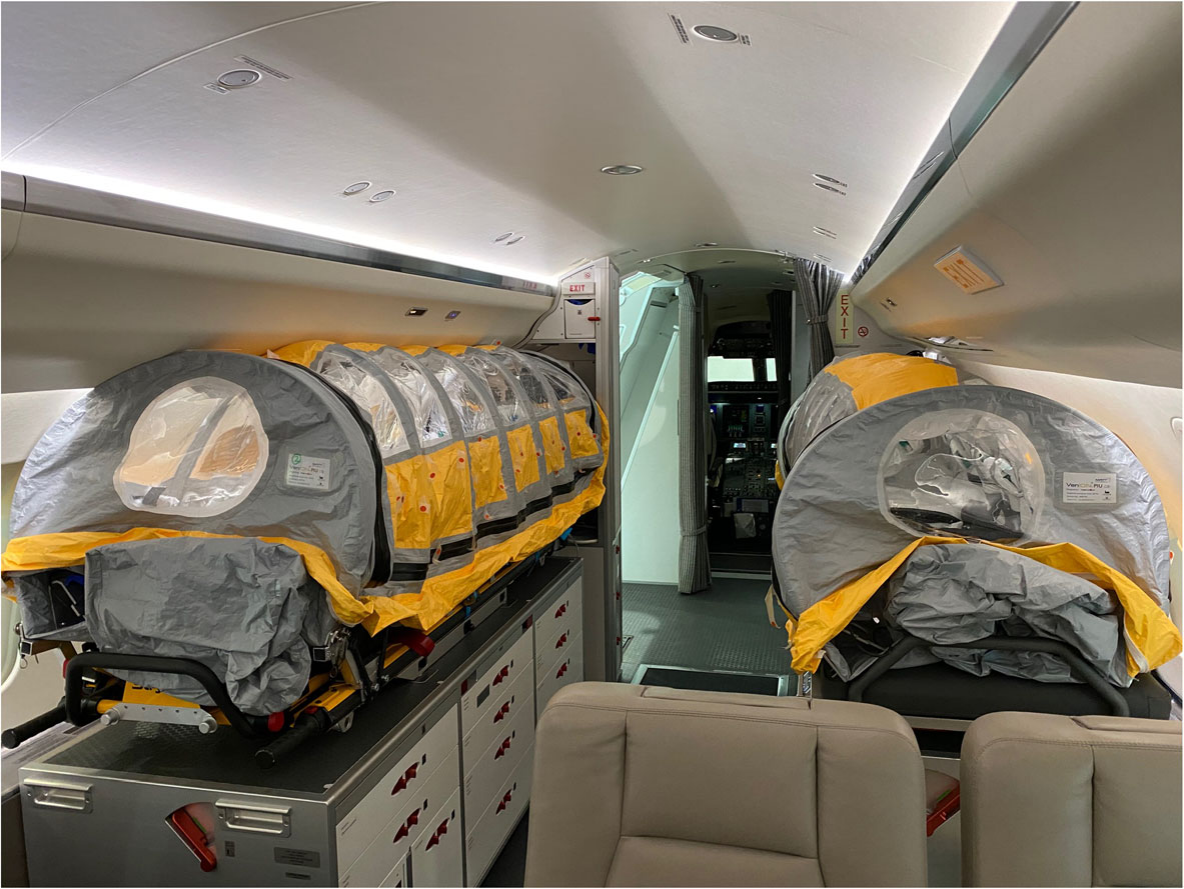
*Two Rega PIUs in the fixed-wing ambulance*

**Fig. 4:**
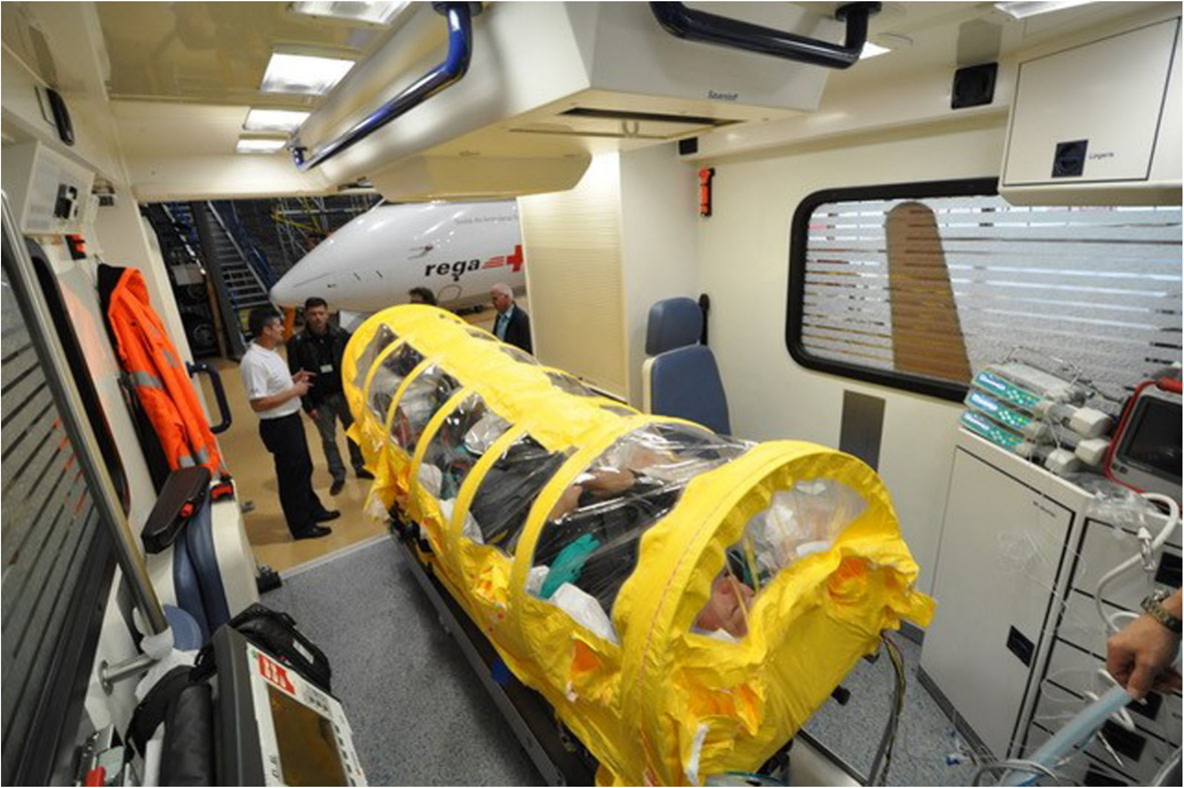
Rega PIU in a ground ambulance

The Rega PIU (Fig. 3) comprises a flexible safety hull stabilized by arched wires mounted on a hard floor plate. It is maintained under negative pressure by a High Efficiency Particulate Air (HEPA) filtered ventilation system that uses aircraft power within the aircraft and battery power when outside, while the cabin pressure is maintained at a standard cabin altitude of 8000 ft. During development, it was successfully tested against infectious agents and liquid penetration using the EN 14126 test method and the EN ISO 17491-3 fixed-wing test, respectively. Altogether, PIU barrier performance proved equal to that provided by protective clothing. Its fixation system allows transportation on any commonly available patient stretcher. The PIU is designed to fit in a fixed-wing ambulance, a medium-sized helicopter, and ground-based ambulances (Fig. 4). Its dimensions are 200 cm*58 cm*63.5 cm. Power for the PIU ventilation is supplied by a rechargeable battery pack (runtime of 8 h) and a 220 V connector.

Albrecht et al. showed in 2015 [6] that a PIU undergoing sudden loss of cabin pressure at a flight level of 30,000 ft. would lose leak tightness. Consequently, the Rega PIU features a built-in “air bag” that allows for additional air volume expansion encountered with sudden loss of cabin pressure [6]. Therefore, it is currently the only available PIU that allows patient transport in pressurized fixed-wing cabins at usual flight levels.

Plazikowski et al. compared airway management in the Rega PIU with airway management under standard protective measures [7]. The study compared intubation of mannequins using a standard laryngoscope, a video laryngoscope, or cricothyrotomy. Overall, intubation of a patient in the PIU was rated subjectively more difficult compared to standard measures (Visual Analogue Scale 76 vs. 9, *p* < 0.01). However, success rates in both groups were comparable. Thus, emergency airway management was shown to be achievable even inside the PIU.

### Rega aeromedical transfer missions involving patients with highly contagious diseases, including COVID-19, since 2015

#### Fixed wing (AEMS) / Rega air ambulance jet CL605

Since 2015, Rega has successfully transferred 16 spontaneously breathing patients and two ventilated patient (COVID-19) in the PIU: one patient with high-risk Ebola virus exposure, four patients with open pulmonary tuberculosis, and 13 patients with confirmed COVID-19. All transports were well tolerated, although in some cases mild sedation was necessary.

In addition, six ventilated patients with COVID-19 were transported without the PIU. All of those patients needed hemodynamic monitoring and two were supported with noradrenaline during the transport. Flight duration ranged between 45 and 699 min.

### Rotary wing (HEMS) / AW 109SP and/or H145

Since the beginning of the current pandemic in Switzerland, in March 2020, eighty-three seriously ill patients (55 men, 28 women) infected with COVID-19 were transported. The majority of patients were classified as NACA 4 or 5 (NACA 4: 30 and NACA 5: 42). Thirty-seven patients were spontaneously breathing, forty-six were intubated and ventilated predominantly in IPPV mode (37 IPPV, 3 CPPV, 6 PCV). Eighteen patients needed noradrenaline support for the transport. Flight duration ranged between 5 and 59 min.

## Conclusion

For primary HEMS missions, SOPs for preventing the transmission of infections during the COVID-19 or other future pandemics need to be based on current recommendations of national medical societies/authorities, adapted to specific circumstances of HEMS missions, and trained in simulation. For secondary aeromedical transport of non-intubated or intubated COVID-19 patients in fixed-wing ambulances, or for long-lasting HEMS missions, the use of small closed PIUs may be beneficial.

## Data Availability

Not applicable.

## Abbreviations

AEMS: Aeroplane Emergency Medical Services
CDR: Commander = pilot
COVID-19: Corona virus disease 2019
CPPV: Continuous positive pressure ventilation
FFP: Filtering face piece
HEMS: Helicopter Emergency Medical Services
IPPV: Ntermittent positive pressure ventilation
NACA: National Advisory Committee for Aeronautics
PCV: Pressure-controlled ventilation
PIU: Patient isolation unit
PPE: Personal protective equipment
SOP: Standard operating procedure
